# Traumatic atlantoaxial rotatory subluxation in an adolescent: a case report

**DOI:** 10.1186/1752-1947-6-27

**Published:** 2012-01-23

**Authors:** Luis Enrique Meza Escobar, Georg Osterhoff, Christian Ossendorf, Guido A Wanner, Hans-Peter Simmen, Clément ML Werner

**Affiliations:** 1 Department of Surgery, Division of Trauma Surgery, University Hospital Zurich, Raemistrasse 100, 8091 Zurich, Switzerland

## Abstract

**Introduction:**

Atlantoaxial rotatory subluxation is rarely caused by trauma in adults. Usually, the treatment of choice is traction using Halo/Gardner-Wells fixation devices for up to six weeks.

**Case presentation:**

We present the case of a 19-year-old Caucasian woman with traumatic atlantoaxial subluxation. Early reduction three hours after trauma and immobilization using only a soft collar were performed and yielded very good clinical results.

**Conclusion:**

In the adult population, atlantoaxial subluxation is a rare condition but is severe if untreated. Early treatment implies a non-surgical approach and a good outcome. Conservative treatment is the recommended first step for this condition.

## Introduction

Atlantoaxial rotatory subluxation is frequently observed in children and in patients with rheumatic arthritis, but rarely occurs traumatically in adults [1]. A typical clinical sign is torticollis [2] with lateral flexion of the neck and contralateral rotation, known as the Cock-Robin position [3]. Usually, the treatment of choice is traction using Halo/Gardner-Wells fixation devices for up to six weeks [4]. The importance of recognizing this condition stems from the fact that it has the potential to cause severe neural damage or even death if it is not treated promptly [5].

We present the case of a patient with traumatic atlantoaxial subluxation in which early reduction, three hours after trauma and immobilization using only a soft collar were performed and yielded very good clinical results.

### Case Presentation

**Figure 1:**
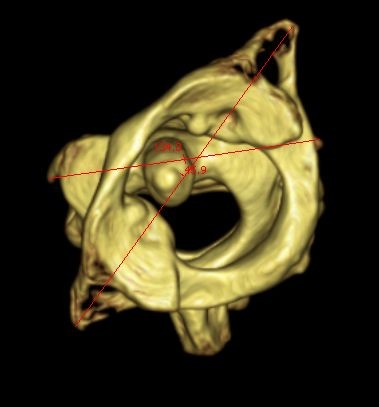
**Atlantoaxial computed tomography scan**. Atlantoaxial rotation of 46° to the left.

**Figure 2:**
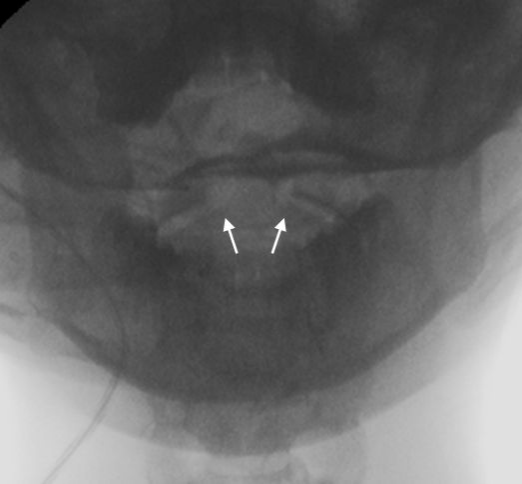
**Transbuccal fluoroscopy after reduction**. Arrows point to the asymmetric distances between the lateral masses of the atlas and the dens axis.

**Figure 3:**
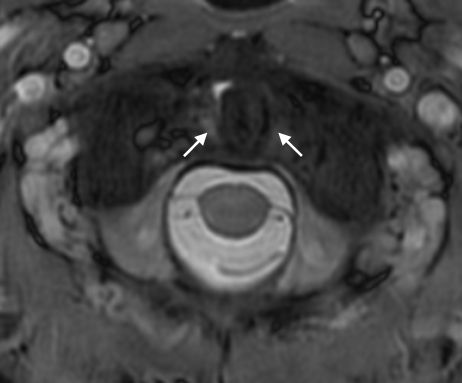
**Atlantoaxial magnetic resonance imaging**. Arrows point to the intact alar ligaments.

**Figure 4:**
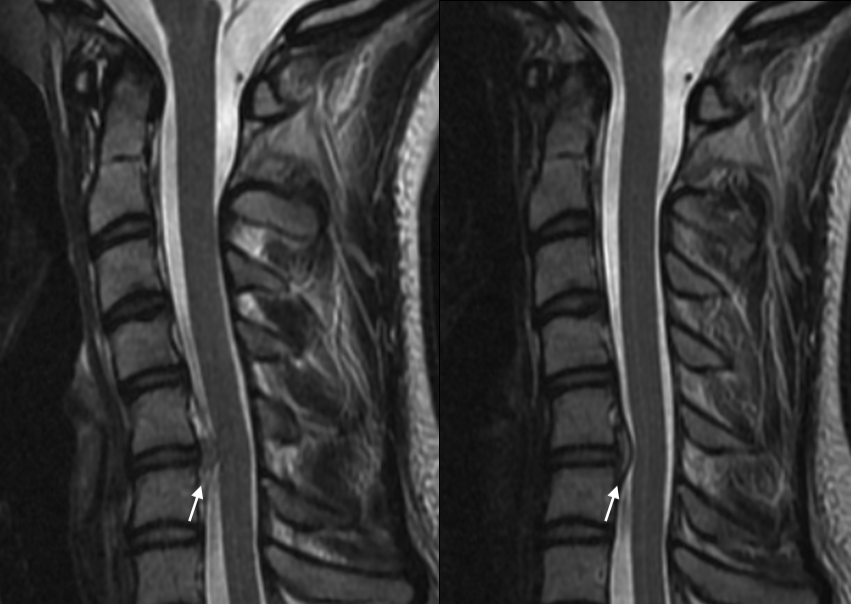
**Sagittal magnetic resonance imaging (T2)**. The magnetic resonance imaging scan of the cervical spine on the day of trauma (**A**) shows an epidural mass (arrows) dorsally to C5/C6 - probably a hematoma or a disc protrusion, without signs of myelopathy. Six weeks later (**B**) the mass has decreased in size, the remaining disc C5/C6 is intact.

**Figure 5:**
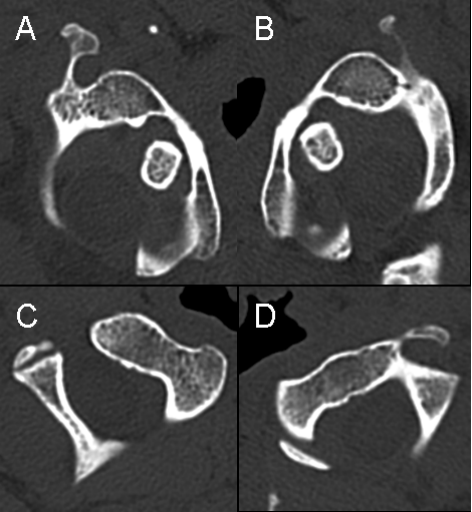
**Rotatory computed tomography scan**. Atlas (**A + B**) and axis (**C + D**) with the head rotated to the left (A + C) and to the right (**B + D**).

While driving a van and wearing a seatbelt, a 19-year-old Caucasian woman, was involved in a head-on vehicle collision (speed about 40 km/hour), followed by a rear-end hit from another vehicle. When rescue services arrived at the scene, the patient was found sitting in her car with her head immobilized in a left rotation. She was transferred onto a spinal board. The application of a stiff neck collar was not possible as her head was fixed in the rotated position. After admittance to a regional hospital, the physician in charge tried to reposition her head but she reported painful paresthesia in the left arm. She was transferred to our spine and trauma center. Upon admittance, the woman complained about strong, immobilizing pain in the upper cervical spine with torticollis to the left side. A computed tomography (CT) scan revealed an atlantoaxial rotation of 46° to the left without any signs of osseous lesions (Figure 1). The neck was then reduced by cautious rotation under traction with the cervical spine in flexion thus avoiding harm by potential posttraumatic disc lesions. During this process, the patient was awake and did not report any new paresthetic sensations during the procedure. There were no clinical signs of neurological sequelae before or after reduction. However, a fluoroscopic control still showed signs of atlantoaxial pathology (Figure 2) and magnetic resonance imaging (MRI) of the cervical spine was done (Figure 3). It showed the integrity of the transverse and the alar ligaments and a traumatic discus protrusion on level C5/6 (Figure 4). After three days of immobilization and analgesic therapy, a CT (with maximum bilateral head rotation) showed no persisting atlantoaxial fixation (Figure 5). Subsequently, she was discharged three days after admittance and immobilized in a soft collar for six weeks. At a follow-up examination six weeks after the trauma, the pain and paresthesia in the left arm had receded completely and the patient had a full range of motion. A follow-up MRI of the cervical spine showed only slight persistent atlantoaxial rotational displacement of C1/2.

## Discussion

The atlantoaxial joint is stabilized in the anteroposterior plane by transverse ligaments and the joint capsule. The alar ligaments pass from the lateral occipital processes to the posterolateral margins of the odontoid apex and their main function is to prevent excessive rotation of this joint. The normal range of rotation is 40 degrees to each side [5]. These rotational movements imply a displacement of C1 over C2, leading to a loss of contact surface between the corresponding facets on each side. In the case of alar ligament disruption, the rotational angle is less than 36 degrees and the contact surface between the facets is less than 60% [6,7]. These are the features that comprise the diagnosis of atlantoaxial subluxation. Therefore, the rotational mismatch between atlas and axis alone is not a valuable parameter to assess the presence of atlantoaxial subluxation and an imaging oriented classification is used.

Atlantoaxial subluxation occurs rarely in the adult population and it is only responsible for 2.5% of all the spinal afflictions [4]. It is predominate in the pediatric population due to an enhanced elasticity of ligaments, horizontally oriented, shallower joint surfaces of the lateral masses, a not fully developed neck musculature and a bigger head-body relationship [8].

Also, conditions that enhance ligamentous laxity such as: Down Syndrome, Morquio Syndrome and Marfan Syndrome, correlate with a higher incidence of rotatory subluxation [9].

The importance of recognizing this condition is the fact that it has the potential to cause severe neural damage, long term sequelae and even death if not treated promptly. The time between the injury and the reduction is crucial as it directly correlates with the prognosis. If untreated after one to three months it becomes irreducible and requires a surgical approach [5,10]. Due to its lower incidence rate, this condition is frequently undiagnosed or the diagnosis is delayed and the outcome is worse [8].

Traditionally, cervical radiography was used to establish a diagnosis. It showed the persistent rotation of the odontoid peg in relation to the lateral masses of the atlas. Currently, the method for diagnosis is the dynamic unenhanced cervical CT scan, usually performed with multiple 1 mm or 3 mm collimation, and post-imaging three-dimensional reconstruction [11]. It allows an easier interpretation, follow-up and classification, according to Fielding and Hawkins [12]:

• Type 1: rotatory subluxation without anterior displacement of the atlas (atlanto-odontal interval ≤3 mm)

• Type 2: rotatory subluxation with anterior displacement of the atlas of 3 mm to 5 mm

• Type 3: rotatory subluxation with anterior displacement of the atlas of > 5 mm

• Type 4: rotatory subluxation with posterior displacement of the atlas.

As mentioned above, the delay between injury and reduction predisposes to the recurrence of this condition and the failure to heal after non-surgical management with the consequent loss of mobility of the upper cervical spine [13].

The management goals of a patient with this condition are to treat the instability of the atlantoaxial joint, restore and prevent possible effects of neurological compromise and to achieve the normal pain-free motion of this joint. Conservative treatment using analgesics, with halter traction or closed reduction maneuvers, is the first step in the treatment of this condition [6,8,13,14].

The decision to take a surgical approach is based on the stability of the joint, its re-dislocation and on the compromise of the transverse alar ligaments. Compared to conservative management, the arthrodesis of the atlantoaxial joint results in a loss of rotation to each side and therefore it is not recommended as the initial treatment [15].

In patients with diagnosed lesions of the cervical spine, concomitant injuries have to be considered. In our case, the patient had an additional epidural hematoma or disc protrusion on level C5/6. This injury might pose a danger to the patient during a closed reduction maneuver if the patient's awareness is impaired. Therefore, it is necessary to perform both CT and MRI before reduction on these patients. In our case, however, the patient was awake and would have been able to report any new paresthetic sensations. There was neither fracture nor instability nor rupture of alar ligaments. This qualified her for conservative management. Some authors suggest treatment with traction and a subsequent halo body jacket for eight to 12 weeks for these patients [8,16].

It has been shown that wearing soft collars produces less motion of the cervical spine in conscious patients [17], even though it would work more likely as a reminder to the patient to restrict his or her own motion [18]. This is especially important to avoid the critical end range rotation.

It was decided that the patient, being very young, and therefore having ligaments of higher elasticity [19], be immobilized in a soft collar for six weeks. To the best of our knowledge this is the first time this treatment has been reported for atlantoaxial subluxation, without the need for halo fixation and while achieving a good clinical outcome.

## Conclusion

Atlantoaxial subluxation is a rare, but severe if untreated, condition in the adult population. The best way to ensure the diagnosis properly is by using a dynamic unenhanced cervical CT with posterior three-dimensional reconstruction. The delay between injury and management affects the prognosis. Early treatment implies a non-surgical approach and a better outcome. Conservative treatment is the first step with this condition.

## Consent

Written informed consent was obtained from the patient for publication of this case report and any accompanying images. A copy of the written consent is available for review by the Editor-in-Chief of this journal.

## Competing interests

The authors declare that they have no competing interests.

## Authors' contributions

EME and GO participated in data research for the case report, designed the figures and drafted the manuscript. CO participated in data research for the case report and in drafting the manuscript. GW and HPS were involved in the surgical decision making and revised the manuscript. CW had the idea for this case report, performed the surgical procedures, was involved in the analysis of the data and revised the manuscript. All authors read and approved the final manuscript.
